# Exploring the Link between Novel Task Proceduralization and Motor Simulation

**DOI:** 10.5334/joc.190

**Published:** 2021-09-27

**Authors:** Ana F. Palenciano, Carlos González-García, Jan de Houwer, Marcel Brass, Baptist Liefooghe

**Affiliations:** 1 Department of Experimental Clinical and Health Psychology, Ghent University, Belgium; 2 Department of Experimental Psychology, Ghent University, Belgium; 3 Mind, Brain, and Behavior Research Center, University of Granada, Spain; 4 Department of Experimental Psychology, Ghent University, Belgium; 5 Berlin School of Mind and Brain, Department of Psychology, Humboldt University of Berlin, Germany; 6 Department of Psychology, Utrecht University, NL

**Keywords:** Cognitive Control, Action, Learning, Working memory

## Abstract

Our ability to generate efficient behavior from novel instructions is critical for our adaptation to changing environments. Despite the absence of previous experience, novel instructed content is quickly encoded into an action-based or procedural format, facilitating automatic task processing. In the current work, we investigated the link between proceduralization and motor simulation, specifically, whether the covert activation of the task-relevant responses is used during the assembly of action-based instructions representations. Across three online experiments, we used a concurrent finger-tapping task to block motor simulation during the encoding of novel stimulus-response (S-R) associations. The overlap between the mappings and the motor task at the response level was manipulated. We predicted a greater impairment at mapping implementation in the overlapping condition, where the mappings’ relevant response representations were already loaded by the motor demands, and thus, could not be used in the upcoming task simulation. This hypothesis was robustly supported by the three datasets. Nonetheless, the overlapping effect was not modulated by further manipulations of proceduralization-related variables (preparation demands in Exp.2, mapping novelty in Exp.3). Importantly, a fourth control experiment ruled out that our results were driven by alternative accounts as fatigue or negative priming. Overall, we provided strong evidence towards the involvement of motor simulation during anticipatory task reconfiguration. However, this involvement was rather general, and not restricted to novelty scenarios. Finally, these findings can be also integrated into broader models of anticipatory task control, stressing the role of the motor system during preparation.

## Introduction

Following instructions is key for our flexible adaptation to changing environments. Generating actions from instructions allows the success at the very first try with a task, in sharp contrast with more time-consuming trial-and-error learning, which mostly drives non-human apes’ behavior (Cole et al., 2013). The behavioral relevance of this skill has motivated a growing body of literature aiming to understand the cognitive and neural mechanisms allowing instructed performance (Brass et al., 2017). In the present study, we aimed to extend these efforts and address whether motor simulation underpins our ability to achieve new tasks using instructions.

Novel instructed performance relies on control mechanisms that exploit the instruction information to prepare us for the upcoming task (Cole et al., 2017, 2018). While both novel (Cole et al., 2017, 2018; Meiran et al., 2015a) and already-known demands (Meiran, 1996; Monsell, 2003) benefit from anticipatory task control, previous research has stressed the role of a particular preparatory mechanism engaged during first encounters with a task: the proceduralization. This process consists of the generation of action-based (or procedural) task representations from novel instructions. In novel task contexts, where no experience has been accumulated yet, these representations are assembled from scratch, by quickly transforming the instruction content from a declarative format into an action-oriented one (Brass et al., 2017). Once the procedural representation is built, it induces a preparedness state in which stimuli reflexively trigger the relevant responses (Hommel, 2000). As a consequence, instruction proceduralization leads to novel actions that are not only fast and efficient but also automatic. Robust evidence supports the presence of the proceduralization process, identifying signatures of instructions-induced automaticity (Meiran et al., 2017). Specifically, the mere encoding of novel stimulus-response (S-R) mappings interferes with the performance in secondary tasks sharing the same stimuli, generating task compatibility effects (González-García et al., 2020; Liefooghe et al., 2012, 2013; Liefooghe & De Houwer, 2018; Meiran et al., 2015a; Meiran & Cohen-Kdoshay, 2012). Nonetheless, despite these results successfully capture the behavioral consequences of the proceduralization, the mechanisms mediating this transformation are uncertain. Thus, an open question in the field is how novel action-based task representations emerge in the absence of any physical experience.

An intriguing possibility is that instruction proceduralization relies on anticipatory motor simulation (Moran & O’Shea, 2020). It has been proposed that the neural system devoted to action-control is not only in charge of overt execution, but also replays (or simulates) actions covertly (Jeannerod, 1994, 2001). Both theoretical (Grush, 2004; Jeannerod, 2001) and empirical work (Guillot & Collet, 2005; Hardwick et al., 2018; Hétu et al., 2013) support that equivalent movements and kinesthetic representations are shared by action execution and simulation. Accordingly, instructions could induce the covert activation of the relevant responses, which could be bound with the stimulus’ one, enabling action-based task coding. This possibility resonates with neuroimaging results showing activity across the motor cortices during novel task preparation (Hartstra et al., 2011, 2012; Ruge & Wolfensteller, 2010). Moreover, it has been recently shown that motor simulation, engaged by imagery, automatizes S-R association processing (Liefooghe et al., 2021) and benefits novel task implementation (Theeuwes et al., 2018). However, in these studies, the mappings are covertly practiced on multiple occasions, whereas instructions-induced automaticity is reported before the first implementation (Meiran et al., 2015a). Furthermore, participants were externally asked to imagine their responses. In consequence, it remains unaddressed whether we engage in motor simulation as a by-default strategy during instruction preparation.

In the current work, we explored the role of motor simulation for novel task proceduralization. Our strategy was to prevent motor simulation while participants encoded novel mappings, by loading the motor system with a finger-tapping task. This dual-task approach follows previous studies in which motor imagery tasks are combined with overt demands to investigate the cognitive processes underpinning motor simulation (Gabbard et al., 2009; Kunz et al., 2009; Stevens, 2005). For instance, Stevens (2005) showed that actions performed in imagery are sensitive to the effector involved in a dual, overt motor task. Imaging running is disrupted by a leg-related motor task, and imaging clapping, by an arm-related motor task (Stevens, 2005). These results stress the overlap between the representations engaged by covert and overt performance, suggesting that the engagement of a particular action representation by motor execution would affect its availability for simulation purposes. In consequence, this opens the possibility to interrogate motor simulation by manipulating the overt action domain (see also Parsons, 1994). Based on this perspective, we designed a paradigm in which the effectors required by novel mappings could overlap or not with those involved in the concurrent finger-tapping. According to the motor simulation framework (Jeannerod, 1994, 2001), the specific action-related representations engaged by the finger-tapping would map into those used for mapping proceduralization, if this process relied on motor simulation. Hence, we predicted an impairment in the proceduralization when the effectors overlapped.

## Experiment 1

In a first experiment, we assessed the impact of concurrent motor demands on novel mapping proceduralization. We used a paradigm where novel S-R mappings were encoded while participants performed a finger-tapping task, and assessed its impact on the first time the mappings were implemented. Our first prediction was to find a general impairment at mapping implementation due to the dual motor demands. To do so, we included a control block, where the S-R mapping task was kept identical, but no finger-tapping was carried out. We expected a better performance in the control block than in the remaining ones, where the finger-tapping was performed. Our second, and more critical hypothesis was to find a magnified impact of the finger-tapping task when it overlapped at the response level with the S-R mappings. This was assessed by comparing two conditions: one in which the same effectors were required by the mappings and the motor task (overlapping response sets), and another one in which an independent set of effectors were required by each task (non-overlapping response sets). We hypothesized an impoverished performance in the overlapping response set condition in comparison with the non-overlapping one.

### Methods

#### Participants

The online study was completed by 100 participants (33 females, 66 males, 1 non-binary individual) on the Prolific Academic Website (*https://www.prolific.co/*). The mean age was 25.36 years old (*SD* = 7.27 years). Participation was compensated with £6 (£5 as a fixed rate and a £1 bonus offered for high performance, but that all participants received). The sample size was set to detect a small effect size (*Cohen’s d* = 0.3) with a 90% power in a paired-sample *t*-test (see *Data Analysis* section).

#### Material

We generated 224 pairs of novel S-R mappings (168 for the experimental procedure, and 56 for the practice sessions) per participant. Each pair consisted of two pictures (two animals, two inanimate objects, or an animal and an inanimate object) located at both sides of the word “index” or “middle”, indicating the relevant fingers to respond. The picture located at the left was linked to a left-hand response with the indicated finger, and the picture at the right, to a right-hand response. Critically, we employed different pictures for every trial, ensuring that the individual S-R associations were always new. In this sense, even when the more general task remained invariant across the experiment (i.e.: to associate the left-side picture with a left-hand response, and the right-side picture, with a right-hand response), the specific S-R associations changed on a trial-by-trial basis, requiring that novel procedural mapping representations were always created.

Pictures were drawn from a database of 1550 images of animate (non-human animals) and inanimate (vehicles and music instruments) objects used in previous studies (Formica et al., 2020; González-García et al., 2020, 2021). All images were in grayscale, with a white background, and centered in a 150*150 pixels square. The response word was typed in Open Sans font, 26 pixels size. The experiment was programmed in JsPsych v.6.1.0 (de Leeuw, 2015).

#### Procedure

**Figure 1:**
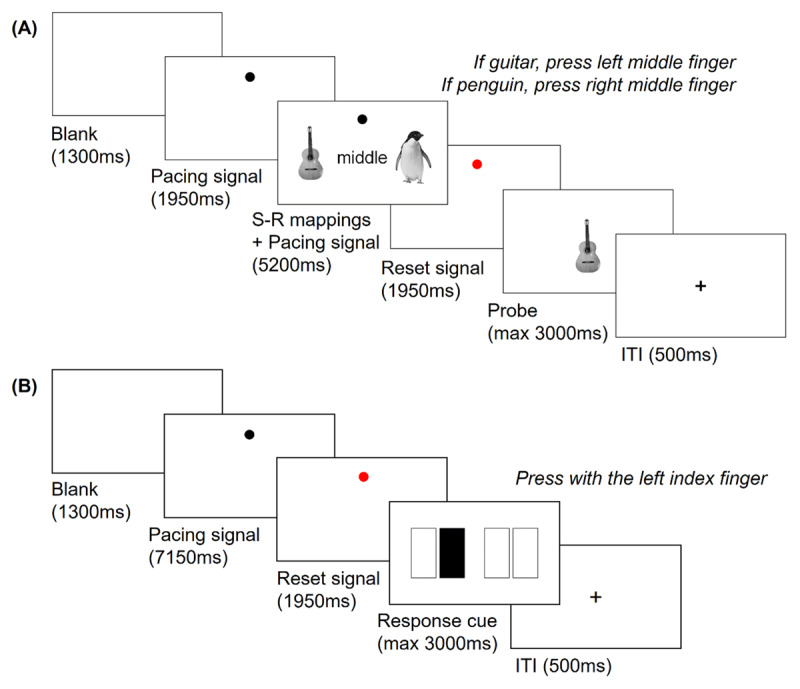
*Experimental paradigms*. **A.** Trial sequence of the paradigm used across Experiments 1–3. **B.** Trial sequence of the paradigm used in Experiment 4.

**Figure 2:**
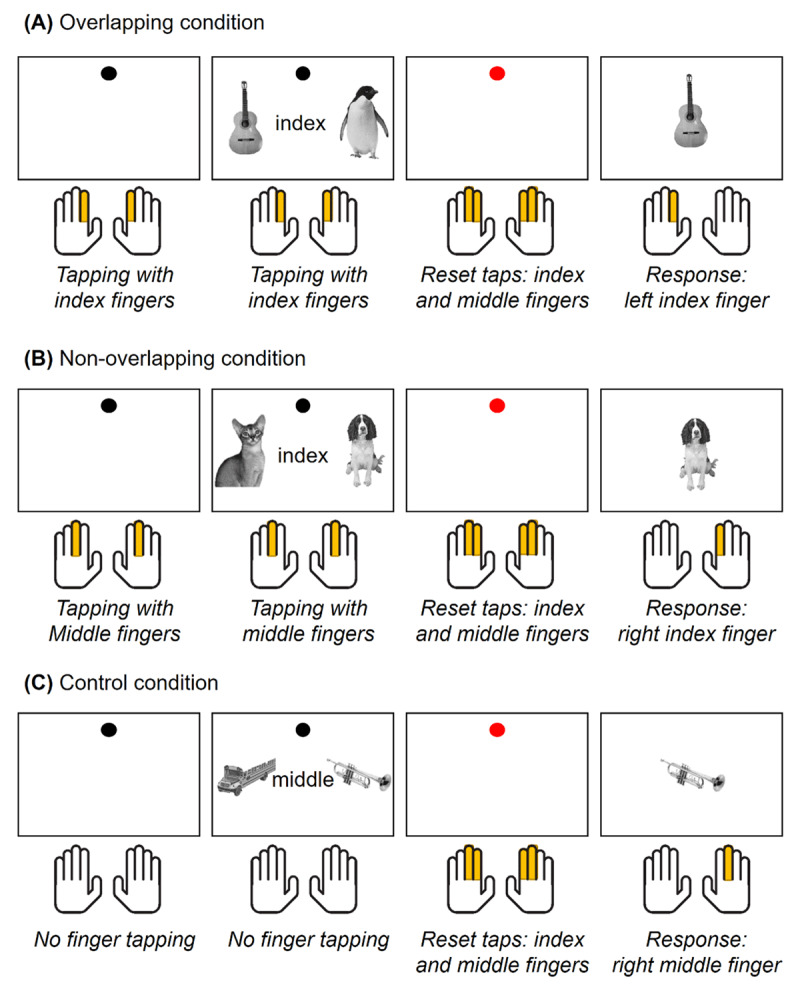
*Experimental conditions from Experiment 1*. **A.** To illustrate the overlapping response set condition, we display a trial from a block in which the index fingers are used for the finger-tapping task. In this trial, an index finger response is also required by the novel S-R mappings, and hence, the response sets overlap between the two tasks. The bottom row shows the responses required by each trial event (finger-tapping task, reset taps, and probe response). **B.** To illustrate the non-overlapping response set condition, we display a trial from a block in which the middle fingers are used for the finger-tapping task. In this trial, an index finger response is required by the novel S-R mappings, and hence, the response sets do not overlap between the two tasks. **C.** In control blocks, no finger-tapping is required during mapping encoding. However, as it is depicted in the bottom row, participants also performed the reset taps and responded to probes.

In each trial, the participants needed to encode and implement novel S-R mappings, while concurrently performing a finger-tapping task (***Figure 1A***). Each trial started with a blank interval (1300 ms) and afterward, a black dot (from now onward, *pacing signal*) appeared rhythmically on the screen at 1.54 Hz. The pacing signal indicated the finger-tapping pace, and participants were instructed to tap every time it flashed on the screen. For each tap, the pacing signal was presented during 100 ms and followed by a blank screen lasting 550 ms (i.e. one tap was required every 650 ms). To entrain the rhythm, participants first tapped three times following the pacing signal before the mappings were shown. Then, the pair of S-R mappings also appeared on the screen, and participants had to memorize them while they kept performing the finger-tapping. The mappings were displayed for 5200 ms, and during this time, the pacing signal flashed eight times. When the encoding time was finishing (3250 ms after the mappings onset), the pacing signal was shown progressively bigger and reddish to warn the participants. Then, the mappings disappeared and a red dot (from now on, *reset signal*) flashed three times on the screen (1.54 Hz, 100 ms of signal followed by 550 ms of blank). This reset signal indicated the participant to tap with both index and middle fingers simultaneously. Reset taps were included to ensure that all fingers were used immediately before responding and to avoid potential response priming effects. Finally, a probe image was presented, and participants had 3000 ms to respond. In ~86% of the trials (regular trials), probes were either the left or the right picture from the mapping. In the remaining ~14% of trials (catch trials), a novel picture was shown, and participants should not respond. Catch trials ensured that both stimuli from the encoding screen were encoded. After the probe, a 500 ms ITI preceded the next trial. The different motor responses required by each trial event are depicted in ***Figure 2A–C***.

We manipulated the overlap between the finger-tapping task and the S-R mappings at the response level (***Figure 2***). To do so, we randomized within blocks the response required by the mappings, either with the index or the middle fingers. Then, we used three modalities of the finger-tapping task in separate blocks: finger-tapping with the index fingers, finger-tapping with the middle fingers, and a control condition without finger-tapping. In the control block, participants were still required to perform the reset taps and to respond to probes (see ***Figure 2C***). This way, we ensured that the only difference with the other conditions was the absence of finger-tapping during mapping encoding. By manipulating the responses required by the mappings and the finger-tapping task, we generated three response set overlap conditions: overlapping response sets (when the same effectors were involved in the mappings and the finger-tapping; ***Figure 2A***), non-overlapping response sets (when different effectors were involved in the mappings and the finger-tapping; ***Figure 2B***), and a control condition (when no finger-tapping was performed during mapping encoding, ***Figure 2C***). Mapping and probe category (animate, inanimate) and response laterality (left, right) were counterbalanced across these three experimental conditions.

Participants completed three blocks, one per finger-tapping modality, of 56 trials each (48 regular trials, 8 catch ones). Information about the finger-tapping modality was provided at the beginning of each block and every 16 trials. At the end of each block, participants saw their mean accuracy rate and could take a pause. Overall, we collected 48 trials per response set overlap condition. All possible block orders were used, ensuring that a balanced number of participants were assigned to each order.

Before the main task, participants completed an extensive three-session practice protocol. Participants were first trained in the S-R mapping task alone, then in the finger-tapping alone, and in a final session, they practiced the two tasks combined. A minimum of 80% correct responses was required to continue with the experimental session. All the mappings used during the practice procedure were never employed during the experiment.

#### Data analysis

We excluded participants with missing data, or whose mean accuracy in the S-R mapping or the motor task (finger-tapping during the encoding period and/or the reset signal) fell below two standard deviations from the sample average. Thirteen participants were excluded from our sample and not further replaced. Within participants, we discarded trials with a reaction time (RT) below or above two standard deviations from their average, and those in which the motor task was not completed (less than seven taps during the encoding period, or less than one reset tap). Catch trials were not included. An average of 9% of trials was excluded per participant. We carried out a one-way repeated-measures ANOVA, with response set overlap (non-overlapping response sets, overlapping response sets, and control) as factor, to explore differences in trial exclusion between conditions. A marginally significant main effect of response set overlap was found, *F*(1.22, 105.10) = 3.473, *p* = .06, η*_p_*^2^ = .04, driven by more excluded trials in the overlapping (*M* = 10%, *SD* = 9%) than in the control condition (*M* = 7%, *SD* = 12%), *t*(86) = 2.53, *p* = .04, *Cohen’s d* = .27. No differences were found between the non-overlapping condition (*M* = 9%, *SD* = 10%) and the rest (non-overlapping vs. control: *t*(86) = 1.91, *p* = .12, *Cohen’s d* = .21; non-overlapping vs. overlapping: *t*(86) = –0.62, *p* = .54, *Cohen’s d* = .04).

Error rates and RT data were analyzed with separates repeated-measures ANOVAs using response set overlap (non-overlapping response sets, overlapping response sets, and control) as a within-subject factor. A Greenhouse-Geisser correction was used whenever sphericity was violated. Planned paired-sample *t*-tests were conducted to address the three pair-wise comparisons between conditions. Despite these comparisons were planned and preregistered, we followed a Bonferroni-Holm correction (Holm, 1979) to control for the multiple comparisons carried out.

Data from the finger-tapping task were analyzed to control for differences in motor performance between the overlapping and non-overlapping response set conditions. We focused on three variables: tapping accuracy (mean percentage of correct taps, computed within trials), tapping delay (averaged taps’ reaction time, taking into account each tap latency regarding its corresponding pacing signal’s onset, and computed within trials), and tapping variability (standard deviation of taps’ reaction time, computed within trials). The three variables were extracted after filtering the data following the approach stated above, and thus, only trials in which the finger-tapping task was substantially performed (above 7 taps) were included. We compared these variables between non-overlapping and overlapping trials with paired-sample *t*-tests. All the analyses were performed using the software JASP (JASP Team, 2020).

For data visualization, 95% confidence intervals were computed after normalizing participants’ data to exclude between-subjects variability (Cousineau, 2005).

### Results

On average, participants responded correctly to probes on 88% of the trials (*SD* = 9%), and the mean RT was 767 ms (*SD* = 272 ms). Participants correctly identified and did not respond to catch probes on an average of 94% of trials (*SD* = 7%). In the finger-tapping task, mean accuracy was 93% (*SD* = 7%), and 77% (*SD* = 11%) of reset taps were performed. Overall, participants understood and fulfilled both the S-R mapping and the finger-tapping demands.

**Figure 3:**
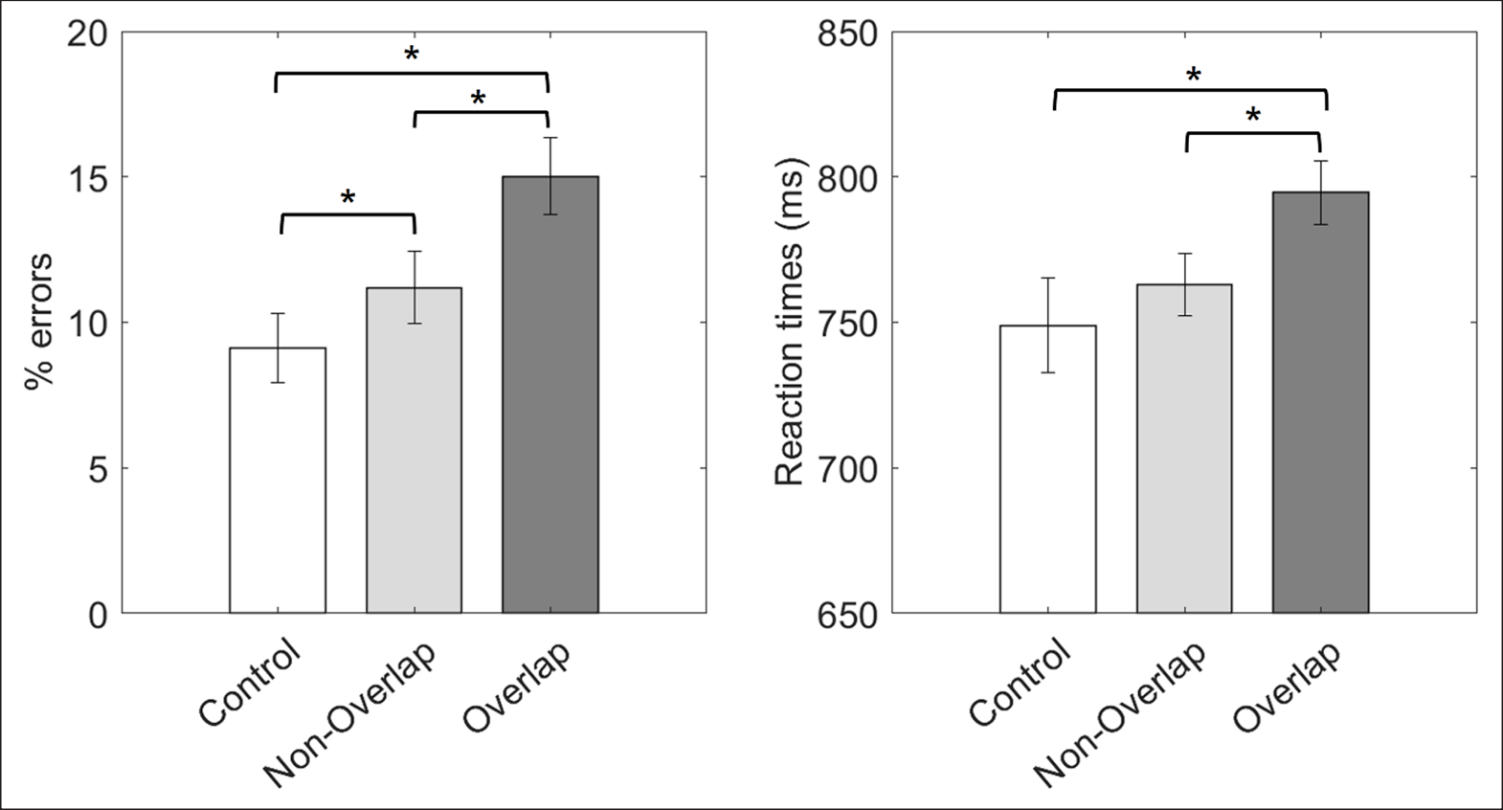
*Results from Experiment 1*. Mean error rate (left) and RT (right) across our three experimental conditions. Asterisks indicate significant differences in the corresponding paired-sample *t*-test (*p* < .05). Error bars display 95% confidence intervals.

Mean error rates and RTs across the three experimental conditions are displayed in ***Figure 3*** and in Supplementary Table 1. The two repeated-measures ANOVAs showed significant main effects in both error rates, *F*(2,172) = 15.02, *p* < .001, η*_p_*^2^ = .15, and RT data, *F*(1.47,126.20) = 8.64, *p* = .001, η*_p_*^2^ = .09. Planned paired-sample *t*-tests showed less errors in the control condition than in both non-overlapping, *t*(86) = 2.03, *p* = .023, *p_corrected_* = .045, *Cohen’s d* = .22, and overlapping trials, *t*(86) = 5.37, *p* < .001, *p_corrected_* < .001, *Cohen’s d* = .58. Responses were also faster in the control than in the overlapping condition, *t*(86) = 3.55, *p* < .001, *p_corrected_* = .001, *Cohen’s d* = .38. No differences in RT were found between control and non-overlapping trials, *t*(86) = 1.10 *p* = .138, *p_corrected_* = .138, *Cohen’s d* = .12. Finally, and more importantly, *t*-tests comparing the non-overlapping and overlapping conditions were significant in both error rates, *t*(86) = 3.26, *p* < .001, *p_corrected_* = .002, *Cohen’s d* = .35, and RT, *t*(86) = 4.46, *p* < .001, *p_corrected_* < .001, *Cohen’s d* = .48, showing better performance in trials were non-overlapping response sets where used.

Finally, as a control analysis, we compared the participants’ engagement in the finger-tapping task between overlapping and non-overlapping trials. Means and standard deviations for tapping accuracy, delay and variability across the two conditions are shown in Supplementary Table 1. *T*-tests showed similar tapping accuracy, *t*(86) = 1.53, *p* = .129, *p_corrected_* = .387, *Cohen’s d* = 0.16, delay, *t*(86) = –1.33, *p* = .186, *p_corrected_* = .387, *Cohen’s d* = –0.14, and variability, *t*(86) = 1.25, *p* = .216, *p_corrected_* = .372, *Cohen’s d* = 0.13 between conditions.

### Discussion

In this experiment, we expected an impairment at mapping implementation due to the dual finger-tapping task, and that this effect would be sensitive to the mappings’ relevant response sets. Confirming our first hypothesis, performance was generally better in the control condition. This effect was robust in error rates, while we could not confirm faster responses in the control than in the non-overlapping condition. This could reflect that in this particular dual-task scenario, where participants’ main task was to assemble procedural representations from scratch, the general dual costs affected processes better captured by error rates. Nonetheless, it is worth noticing that in previous literature, dual-task costs have been typically reported in response speed (Pashler, 1994). Alternatively, this result could also relate to less reliable RTs in control trials (see ***Figure 3***, error bars) due to the absence of finger-tapping, which could have entrained participants’ responses in the other conditions. While no robust conclusions can be drawn in this regard, we can nonetheless confirm that the dual motor demands had a general impact on mapping implementation.

More importantly, we also confirmed our second hypothesis. Performance was slower and more prone to errors in the overlapping than in the non-overlapping condition. In this regard, we ruled out that the finger-tapping facilitated or primed the overlapping fingers’ responses. The inclusion of reset taps before the probe, together with the exclusion of trials in which such reset taps were not performed, ensured that overlapping and non-overlapping fingers were equally primed before responding. Moreover, if response priming had obscured our results, we should have found opposite results. Hence, a priming-based account seems unlikely. Alternatively, a differential engagement in the finger-tapping task could have also contaminated our findings, if participants had performed the tapping to a lesser extent in the non-overlapping condition. To avoid that, we excluded trials in which the finger-tapping was substantially not performed. To control for more subtle differences, we also analyzed tapping accuracy and rhythmicity, not finding differences between overlapping conditions. Thus, an explanation based on finger-tapping performance was ruled out.

Overall, this dataset showed that novel mapping implementation was affected by concurrent motor demands, especially when the response sets engaged by the mapping and the finger-tapping overlapped. This pattern suggested that the proceduralization may entail anticipatory motor simulation, hindered in the overlapping condition. Nonetheless, other cognitive processes also engaged during instructions implementation, as the declarative encoding of the mappings (Brass et al., 2017), could be also the source of our findings. Hence, we carried out a second experiment to directly test that the overlap manipulation disrupted the proceduralization.

## Experiment 2

To clarify the link between the response set overlap manipulation and the proceduralization, we next manipulated the necessity to prepare in advance the novel mappings. We assumed that the proceduralization would be stronger for mappings better prepared (Liefooghe et al., 2013; Meiran et al., 2015a). The preparation demands were varied by using different response deadlines. Under a more restrictive, early response deadline, participants needed to prepare the mappings to a higher degree to be able to respond to probes in a shorter time window. These preparation demands were lower in a more relaxed, late deadline condition. Similar approaches have been used in the past to manipulate task preparation during novel (Liefooghe et al., 2013) and practiced (Lien et al., 2005) mapping implementation.

We adapted our paradigm to include the two response deadlines. Since the previous experiment already provided evidence about the general motor demands cost, we excluded the control condition, and finger-tapping was performed in all blocks. First, we expected to replicate the response set overlap effect, i.e., more errors and slower responses in overlapping than non-overlapping response set trials. Second, we predicted that the response set overlap would interact with the response deadline, with an increased impairment of overlapping response sets under the early response deadline – where higher preparation demands were imposed.

### Methods

#### Participants

The online study was completed by 92 participants (36 females, 55 males, 1 non-binary individual). The mean age was 25.56 years old (*SD* = 9.00 years old). All participants received an economic compensation of £6 (£5 as a fixed rate and a £1 bonus offered for high performance, but that all participants received). The sample size was set to detect a small interaction effect (*Cohen’s d* = 0.2) with 90% power in a repeated-measures ANOVA (see *Data analysis* section).

#### Material

260 pairs of S-R mappings were created per participant, using the same stimuli and procedure as in Experiment 1. We assigned 54 of the mappings to the practice sessions, and the remaining 208, to the experimental task.

#### Procedure

We used the paradigm from Experiment 1, with two modifications. First, the probe’s maximum duration was set to 2000 ms in late response deadline blocks and adapted to each participant’s performance in early response deadline blocks, using data from the initial practice procedure. Second, we provided feedback after each trial. The words “Correct!”, “Wrong!” or “Too slow!” appeared 500 ms after the participants’ response. Slow response feedback was used only during the early deadline blocks.

To compute the early response deadline, we focused on the third practice session, in which participants were trained with the S-R mapping task in combination with the finger-tapping (see *Experiment 1 – Procedure*). Participants completed 20-trial blocks until they achieved an 80% accuracy. The early deadline was adjusted to the mean RT from correct trials during the last practice block. The mean early deadline used was 867 ms (*SD* = 257 ms), ranging from 326 ms to 1418 ms.

The experimental task consisted of four blocks of 52 trials each (48 regular trials, 4 catch ones). Participants completed two blocks per response deadline, one using the index fingers for the motor task, and another using the middle fingers. The response required by the mappings (index, middle fingers), and as a consequence, the response set overlap (overlapping, non-overlapping response sets) were randomized within blocks. We arranged blocks according to the response deadline, with participants completing first the two early deadline blocks and then the two late deadline ones, or vice-versa. We pseudorandomized block order regarding the finger-tapping modality. At the beginning of the block and every 13 trials, the motor task and deadline condition were presented on the screen. Overall, we collected 48 trials per experimental condition.

#### Data analysis

We used the same criterion as in Experiment 1 to exclude participants, discarding data from nine participants. Due to unequal RT distributions, trial trimming was performed independently for each response deadline condition, excluding trials with an RT two standard deviations above or below the condition’s average. Trials in which the finger-tapping was not performed (less than seven taps during the encoding period, or less than one reset tap) were also discarded. An average of 8% (*SD* = 6%) trials were excluded per participant. We carried out a repeated-measures ANOVA, with response set overlap (non-overlapping, overlapping) and response deadline (early, late) as factors, to explore differences in trial exclusion across conditions. The main effect of response set overlap was significant, *F*(1,82) = 6.34, *p* = .014, η*_p_*^2^ = .07, reflecting that more trials were rejected in the overlapping (*M* = 8%, *SD* = 6%) than in the non-overlapping condition (*M* = 7%, *SD* = 6%). Neither the main effect of response deadline, *F*(1,82) = 6.34, *p* = .014, η*_p_*^2^ = .07 (early: *M* = 8%, *SD* = 8%; late: *M* = 8%, *SD* = 7%), nor the interaction were significant.

To address our main hypothesis, we ran repeated-measures ANOVAs on error rates and RT data using response set overlap (non-overlapping, overlapping) and response deadline (early, late) as within-subjects factors. Planed comparison included paired-sample *t*-tests contrasting non-overlapping and overlapping trials separately for early and late response deadline blocks. In all further exploratory analyses, a Bonferroni-Holm correction for multiple comparisons (Holm, 1979) was used.

### Results

**Figure 4:**
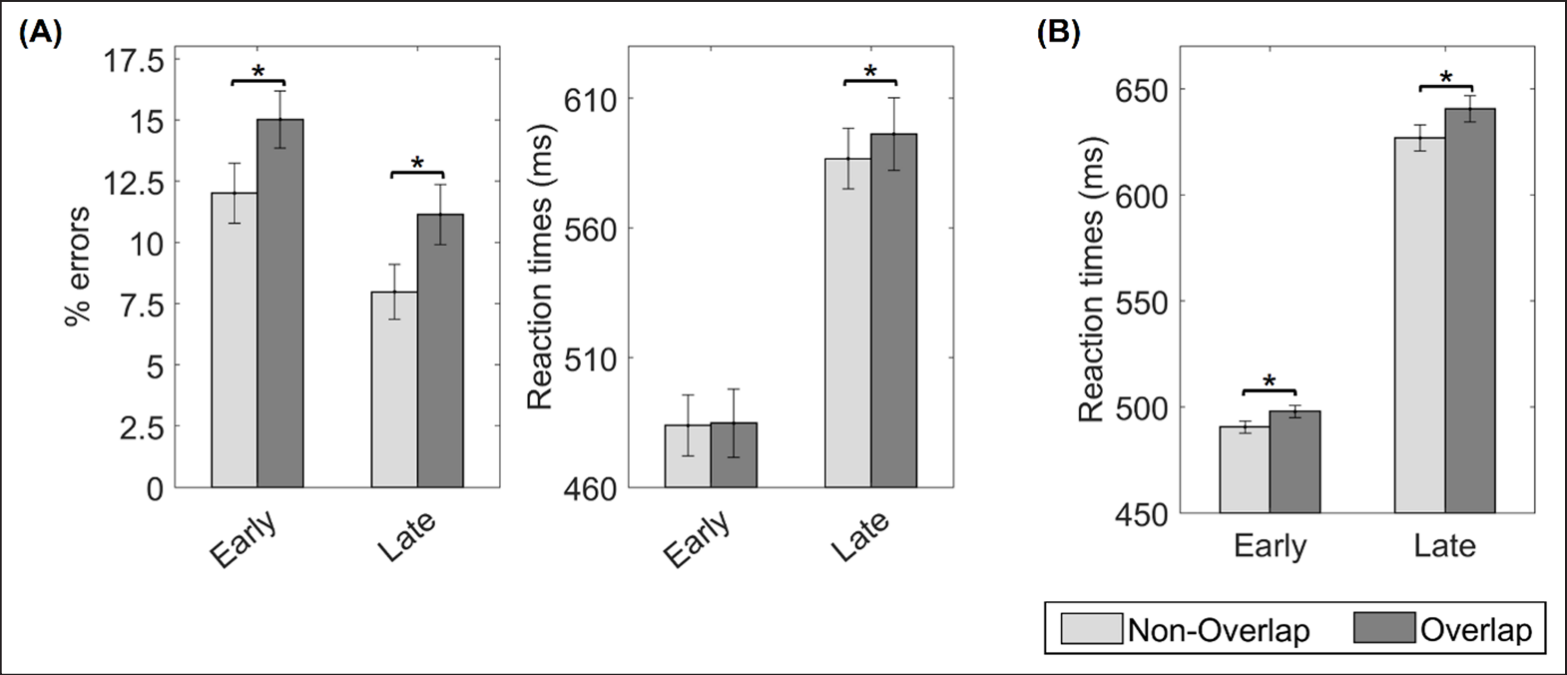
*Results from Experiment 2*. **A.** Mean error rate (left) and RT (right) for non-overlapping and overlapping trials in the early and late deadline conditions. **B.** Averaged RTs from the first two blocks and including the response deadline condition as a between-subject factor. Asterisks indicate significant differences in the corresponding paired-sample *t*-test (*p* < .05). Error bars display 95% confidence intervals.

Mean error rates and RTs across conditions are displayed in ***Figure 4A*** and in Supplementary Table 2. The repeated-measures ANOVA on error data showed a significant main effect of response set overlap, *F*(1,82) = 16.57, *p* < .001, η*_p_*^2^ = 0.17, and response deadline, *F*(1,82) = 25.31, *p* < .001, η*_p_*^2^ = 0.24. Participants committed more errors in the overlapping than in the non-overlapping condition. The error rates were also higher in the early response deadline than in the late one. However, the interaction term was not significant, *F*(1,82) = 0.03, *p* < .875, η*_p_*^2^ < 0.01. Planned comparisons (***Figure 4A***, left panel) showed less errors to non-overlapping than to overlapping mappings in both early, *t*(82) = 3.30, *p* = .001, *Cohen’s d* = 0.36, and late response deadline conditions, *t*(82) = 3.62, *p* < .001, *Cohen’s d* = 0.40

In RT data, we also found a significant main effect of response set overlap, *F*(1,82) = 4.68, *p* = .033, η*_p_*^2^ = 0.05, with faster responses in the non-overlapping than in the overlapping condition. As expected, response deadline was also significant, *F*(1,82) = 75.69, *p* < .001, η*_p_*^2^ = 0.48, with faster responses with the early response deadline than with the late one. Finally, we found a tendency toward a significant interaction, *F*(1,82) = 3.39, *p* = .069, η*_p_*^2^ = 0.04. Following our preregistered analyses, we assessed the effect of response set overlap within each response deadline condition (***Figure 4A***, right panel). In late response deadline blocks, responses were faster in non-overlapping than in overlapping trials, *t*(82) = 2.33, *p* = .022, *Cohen’s d* = 0.26. We did not find evidence supporting this effect in early response deadline blocks, *t*(82) = 0.38, *p* = .708, *Cohen’s d* = 0.04,

Further exploratory analyses addressed whether the order in which the response deadlines were experienced entailed a carry-over of the preparation strategy across blocks. To do so, we ran repeated-measures ANOVAs on errors and RT data with response set overlap and response deadline as within-subjects factors, and response deadlines’ order (early-late, late-early) as between-subject factor. In error rates, neither the main effect of the response deadlines’ order, *F*(1,81) = 2.45, *p* = .121, η*_p_*^2^ = .03, nor its interaction with other terms were significant (response deadlines’ order * response set overlap: *F*(1,81) = 3.17, *p* = .08, η*_p_*^2^ = 0.04; response deadlines’ order * response deadline:, *F*(1,81) = 0.71, *p* = .401, η*_p_*^2^ = 0.01; three-way-interaction: *F*(1,81) = 0.64, *p* = .426, η*_p_*^2^ = 0.01). The main effects of response set overlap, *F*(1,81) = 16.07, *p* < .001, η*_p_*^2^ = 0.17, and response deadline, *F*(1,81) = 25.64, *p* < .001, η*_p_*^2^ = 0.24, remained significant, indicating a stable pattern irrespectively of block order. In RT data, however, we found a significant interaction between the response deadline and the response deadlines’ order, *F*(1,81) = 24.94, *p* < .001, η*_p_*^2^ = 0.24, and also a significant three-way-interaction, *F*(1,81) = 5.35, *p* = .023, η*_p_*^2^ = 0.06. Posthoc comparisons showed that for participants performing first the early response deadline, a trend towards faster responses in non-overlapping than overlapping trials was found in early deadline blocks, *t*(38) = 2.54, *p* = 0.015, *p_corrected_* = 0.06, but not in the late deadline ones, *t*(38) = 0.90, *p* = .377, *p_corrected_* = 0.396. Conversely, for participants performing first the late response deadline condition, a trend toward the effect of response overlap was found in the late deadline blocks, *t*(43) = 2.26, *p* = .029, *p_corrected_* = 0.087, but not in early deadline ones, *t*(43) = –1.31, *p* = .198, *p_corrected_* = 0.396).

Next, to avoid the potential carry over-effect, we focused on the first half of the experiment, in which only one of the response deadlines was used. The first two blocks’ RT data were analyzed in an ANOVA with response set overlap as a within-subjects factor, and response deadline as a between-subjects factor. Mean RTs across conditions from the two first blocks are shown in ***Figure 4B***. We found significant main effects of response set overlap, *F*(1,82) = 9.02, *p* = .004, η*_p_*^2^ = 0.10, and response deadline, *F*(1,82) = 21.03, *p* < .001, η*_p_*^2^ = 0.21. Nonetheless, the interaction term was not close to significance in this analysis, *F*(1,82) = 0.85, *p* = .360, η*_p_*^2^ = 0.01.

Finally, we analyzed finger-tapping performance with separate repeated-measures ANOVAs for tapping accuracy, variability, and delay, using response set overlap and response deadline as within-subject factors. The three variables’ mean and standard deviation across conditions are shown in Supplementary Table 2. No significant main or interaction effects were found in these ANOVAs (all *F* values < 1, all *p* values > .5).

### Discussion

Experiment 2 replicated the response set overlap effect found in the first dataset. When overlapping response sets were used, participants committed more errors and responded slower. However, the data did not support our prediction that the overlap effect was heightened under high-preparation demands. Error rates were equally affected by the response set overlap under the two response deadlines. RT data showed a tendency toward a significant interaction, which nonetheless went in the opposite direction, with a greater overlap effect in the late response deadline blocks.

Further exploratory analyses showed that RT data could reflect a carry-over of the preparatory strategy between response deadline conditions. First, when the response deadlines’ order was included in the analysis, it modulated the effect of the response set overlap. Second, when only data from the first half of the experiment (when one response deadline was applied) was analyzed, the response set overlap affected equally both response deadline conditions. Thus, these results call for extra caution when interpreting the tendency toward an interactive pattern found in RT.

More critically, the response deadlines induced a speed-accuracy trade-off, with the early response deadline increasing the response speed, but at the cost of more errors. This result contrasts with the benefit in both accuracy and RT previously reported (Liefooghe et al., 2013), and could indicate that our manipulation was not optimal in inducing differential preparatory strategies. As a consequence, it is difficult to infer how the early response deadline affected the participants’ performance. The error rate results and the exploratory RT analyses supported a response set overlap effect in this condition, suggesting that mappings were prepared following a similar simulation strategy as with the late response deadline. Nonetheless, the observed speed-accuracy trade-off could also reflect that the response time constraints induced a non-optimal preparatory strategy or an impoverished probe processing. This ambiguous pattern could have been caused by the early response deadline used here, computed from a reduced amount of trials. More sophisticated, but also more time-consuming calibration procedures may have generated a response deadline better fitted to individual performance. Here, we aimed to find a compromise between the overall experiment duration and adapting the response deadline to our participants. However, this may have hindered our capacity to manipulate task preparation.

Overall, Experiment 2 successfully replicated the response set overlap effect. However, we did not confirm the hypothesized enhancement of this effect under stringent preparation demands. This may reflect that the finger-tapping task did not interfere with task preparation in general, nor with the proceduralization in particular. Nonetheless, taking into account the speed-accuracy trade-off induced by the deadline procedure, we believe that is more cautious to conclude that we did not succeed in inducing different preparatory strategies. In consequence, this dataset was inconclusive regarding the relationship between the overlap effect and proceduralization.

## Experiment 3

To overcome the previous limitations, we carried out a third experiment using a more direct manipulation of task proceduralization: the novelty of the S-R mappings. Comparing novel and practiced instructions is a common manipulation in the instructed-behavior literature to isolate the proceduralization process (Brass et al., 2017; Cole et al., 2013). For novel S-R associations, the procedural task set must be quickly assembled from scratch. Practiced task sets, on the contrary, can be directly retrieved from long-term memory, bypassing the proceduralization (Meiran et al., 2012). Thus, while both novel and practiced tasks are prepared in advance, the processing chain differs, with new mappings additionally requiring their proceduralization. Based on this view, we included in our paradigm new and multiple-times-applied mappings. We predicted that the interference due to overlapping response sets would be greater for the novel mappings, supporting the link between motor simulation and novel task proceduralization.

### Methods

#### Participants

Ninety-two participants (38 females, 53 males, 1 non-binary gender participant) completed the online experiment. The mean age was 28.58 years old (*SD* = 9.59 years old). Participants received an economic compensation of £6 (a £5 fixed rate, and a £1 bonus offered for high performance, but that all participants received). The sample size was set to detect a small interaction effect (*Cohen’s d* = 0.2) with 90% power in repeated-measures ANOVAs (see *Data analysis* section).

#### Material

Ninety-six pairs of S-R mappings were created per participant. Mappings were composed of pictures of either two animate or two inanimate objects, associated either with an index or middle fingers response. Eight mappings were assigned to the practiced condition, and the remaining 88, to the novel one. The eight practiced mappings were split into two sets, one per motor task modality (index, middle finger-tapping). Each practiced mapping set was the result of crossing the two possible categories (animate, inanimate) and responses (index, middle fingers).

#### Procedure

We use the paradigm from Experiment 1 (***Figure 1A***), but now including both novel and practiced S-R mappings. All practiced mappings were learned during a previous practice protocol (see below).

The experiment consisted of four blocks, of 44 trials each (40 regular and 4 catch trials). Participants completed two blocks with novel mappings, and another two repeating the same set of learned mappings. Within each novelty condition, participants fulfilled one block per finger-tapping modality (index, middle fingers). In the practiced blocks, independent subsets of mappings were used for each finger-tapping modality. Within blocks, we randomized the response required by the mappings (index, middle fingers) and in consequence, the response set overlap condition (overlapping, non-overlapping response sets). Block order was arranged according to the novelty manipulation, with participants first fulfilling two novel blocks and then two practiced ones, or vice-versa. Block order was pseudorandomized regarding the finger-tapping modality. At the beginning of each block, and every 11 trials, participants read the mapping novelty and finger-tapping conditions. Overall, we collected 40 trials per experimental condition.

The eight practiced mappings were learned during the initial practice protocol (see *Experiment 1 – Procedure*). In the first and the third session, participants repeatedly implemented the practiced S-R mappings, alone and combined with the finger-tapping task, respectively. Across these two sessions, each mapping was presented at least eight times.

#### Data analysis

We used the same criteria as in Experiment 1 for participant and trial exclusion. Data from eight participants were discarded. Within participants, we excluded an average of 7% (*SD* = 3%) of trials. To identify differences on trial exclusion among conditions, we ran a repeated-measures ANOVA using response set overlap (non-overlapping, overlapping response sets) and mapping novelty (novel, practiced) as factors. Neither the main effects (response set overlap: *F*(1,83) = 1.62, *p* = .206, η*_p_*^2^ = 0.02, mapping novelty: *F*(1,83) = 0.10, *p* = .748, η*_p_*^2^ < 0.01) nor the interaction term, *F*(1,83) = 0.18, *p* = .676, η*_p_*^2^ < 0.01, were significant, showing that an equivalent amount of trials were excluded across conditions (non-overlapping: *M* = 7%, *SD* = 4%; overlapping: *M* = 7%, *SD* = 4%; novel: *M* = 7%, *SD* = 5%; practiced: *M* = 7%, *SD* = 6%).

We conducted repeated-measures ANOVAs with response set overlap (non-overlapping, overlapping response sets) and mapping novelty (novel, practiced) as factors, on error rates and RT data. Planned comparison included paired-sample *t*-tests to contrast between non-overlapping and overlapping response set trials, separately for novel and practiced mappings.

### Results

**Figure 5:**
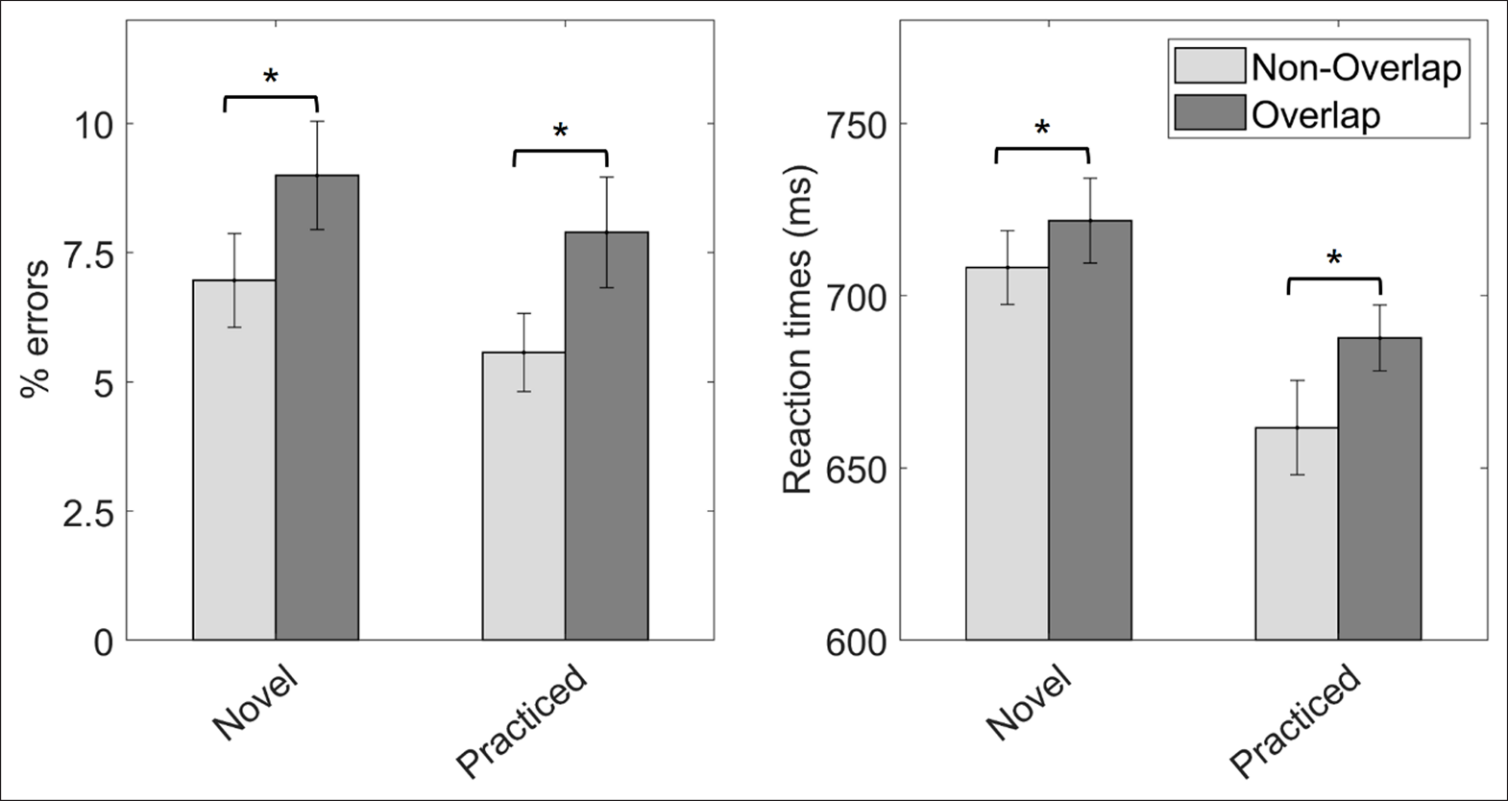
*Results from Experiment 3*. Mean error rate (left) and RT (right) for non-overlapping and overlapping trials in the novel and the practiced mapping conditions. Asterisks indicate significant differences in the corresponding paired-sample *t*-test (*p* < .05). Error bars display 95% confidence intervals.

Mean error rates and RTs across experimental conditions are displayed in ***Figure 5*** and in Supplementary Table 3. In the error rate repeated-measures ANOVA, we found a significant main effect of response set overlap, *F*(1,83) = 16.87, *p* < .001, η*_p_*^2^ = 0.17, driven by more errors in the overlapping than the non-overlapping condition. The main effect of mapping novelty was close to significance, *F*(1,83) = 3.69, *p* = .058, η*_p_*^2^ = 0.04, with numerically higher error rates in the novel condition. The interaction term was non-significant, *F*(1,83) = 0.10, *p* = .754, η*_p_*^2^ < 0.01. Planned comparisons (***Figure 5***, left panel) confirmed that response set overlap affected both novel, *t*(83) = 2.74, *p* = .008, *Cohen’s d* = .30, and practiced mappings, *t*(83) = 3.46, *p* < .001, *Cohen’s d* = .38.

In RT data, the main effects of response set overlap, *F*(1,83) = 12.20, *p* < .001, η*_p_*^2^ = 0.13, and mapping novelty, *F*(1,83) = 17.88, *p* < .001, ηp2 = 0.18, were significant. Participants were faster responding in non-overlapping than in overlapping trials, and to practiced than novel mappings. The interaction term was not significant, *F*(1,83) = 2.60, *p* = .111, η*_p_*^2^ = 0.03. Planned comparisons (***Figure 5***, right panel) showed a significant overlap effect for both practiced, *t*(83) = 3.66, *p* < .001, *Cohen’s d* = .50, and novel mappings, *t*(83) = 2.06, *p* = .043, *Cohen’s d* = .23.

Numerically, the effect of response set overlap on RT was greater for practiced instead of novel mappings (see ***Figure 5***). To explore this finding, we analyzed performance during practiced blocks and assessed whether the overlap effect was sensitive to the experience accumulated during the time-on-task. Each practiced mapping was implemented ten times during the experiment. We split our data between the first five and the last five repetitions and ran a repeated-measures ANOVA with response set overlap and mapping repetition (first five, last five) as within-subject factors on RTs of practiced blocks. As expected, the main effect of response set overlap was significant, *F*(1,83) = 13.76, *p* < .001, η*_p_*^2^ = .14. Mapping repetition was also significant, *F*(1,83) = 6.92, *p* = .010, η*_p_*^2^ = .08, with response speed improving across the experiment (First half: *M* = 683 ms, *SD* = 238 ms; Last half: *M* = 667 ms, *SD* = 377 ms). The interaction was, however, non-significant, *F*(1,83) = 0.01, *p* < .940, η*_p_*^2^ < .001.

Finally, we analyzed finger-tapping performance. Descriptive statistics from the three finger-tapping variables analyzed are displayed in Supplementary Table 3. Repeated measures ANOVAs, with response set overlap and mapping novelty as factors, showed equivalent tapping accuracy and variability across experimental conditions. Neither the main effect of response set overlap (tapping accuracy: *F*(1,83) = 0.63, *p* = .429, η*_p_*^2^ < .01; tapping variability: *F*(1,83) = 1.55, *p* = .216, η*_p_*^2^ = .02), the main effect of mapping novelty (tapping accuracy: *F*(1,83) = 1.28, *p* = .261, η*_p_*^2^ = .02; tapping variability: *F*(1,83) = 2.24, *p* = .138, η*_p_*^2^ = .03) nor the interaction term (tapping accuracy: *F*(1,83) = 1.36, *p* = .248, η*_p_*^2^ = .02; tapping variability: *F*(1,83) = 2.46, *p* = .121, η*_p_*^2^ = .03) were significant. Regarding tapping delay, we found a tendency toward a significant main effect of mapping novelty, *F*(1,83) = 3.61, *p* = .061, η*_p_*^2^ = .04, indicating shorter tapping delays during novel mapping encoding. The main effect of response set overlap, *F*(1,83) = 1.80, *p* = .183, η*_p_*^2^ = .02, and the interaction term, *F*(1,83) = 0.56, *p* = .458, η*_p_*^2^ < .01, were not significant.

### Discussion

In Experiment 3, we aimed to assess the impact of the overlapping motor demands on the proceduralization process, by comparing this effect across new and practiced S-R associations (e.g. Cole et al., 2018). We predicted a magnified overlap effect linked to novelty. The current dataset further replicated the previous experiments, showing an impoverished mapping performance in overlapping response set trials. The hypothesized interaction with task novelty was, however, not found. Finally, we explored whether the experience accumulated with the practiced mappings along the experiment modulated the overlapping effect. However, the overlap effect was constant over time.

Overall, we did not evidence a differential impact of response set overlap depending on novelty. This null result could be related to the practiced condition that we used, generated by repeating a set of mappings at least eight times before starting the experiment. Theoretically, S-R associations change their status after the first implementation, when the procedural task set can be traced into long-term memory (Meiran et al., 2015a). From this view, eight repetitions should suffice to differentiate novel and practiced associations. Having said that, previous empirical works employed more extensive practice procedures (e.g. Cole et al., 2018; González-García et al., 2017). This leaves open the possibility that our distinction between novel and practiced tasks was less salient due to lack of experience.

Nonetheless, it seems more plausible that task novelty was indeed unrelated to our overlap manipulation. This implies that the detrimental effect caused by the finger-tapping was mediated by a mechanism common to novel and practiced task settings. On one hand, it could be a preparatory-related process, as the maintenance of the procedural task set, or more downstream motor planning. On the other, our results could be caused by preparatory-unrelated mechanisms. To decide between these accounts, we ran a final experiment.

## Experiment 4

Experiments 1–3 replicated the effect of response set overlap. However, when we attempted to better characterize its significance for novel task proceduralization, we obtained inconclusive evidence. This opens the possibility that instead of disrupting task preparation, the impact of our effect relies on pure motor processes. The intensive and repetitive finger-tapping could have generated a negative priming effect, with impoverished responses to probes with the effectors primed by the finger-tapping. Similarly, effectors’ fatigue could operate in the same direction. Both accounts predict the detrimental effect of overlapping response sets. To test these possibilities, we conducted a fourth experiment in which the response set overlap effect was assessed in the absence of task preparation. Participants performed the finger-tapping task without further demands, to later respond to explicit, instructional cues indicating the appropriate key press. The cues’ responses overlapped or not with the ones required by the finger-tapping. Since no preparation was required during the finger-tapping, a significant overlap effect would indicate that our findings were driven by motoric processes. Contrary, observing a null effect would support that the overlap actually affected anticipatory task control.

### Methods

#### Participants

We collected data from 92 participants (34 women, 58 men). The mean age was 27.40 years old (*SD* = 8.57 years old). Participants received an economic compensation of £3.5 (a £3 fixed rate, and a £0.5 bonus offered for high performance, but that all participants received). The sample size was set to detect a small effect size (*Cohen’s d* = 0.3) with a 90% power in paired-sample *t*-tests (see *Data Analysis* section).

#### Material

We used four cues that explicitly indicated the required response. The response cues were composed of four rectangles (150 × 75 pixels) located along the horizontal axis of the screen. Each rectangle corresponded to a finger: from left to right, to the left middle, left index, right index, and right middle finger. The rectangle indexing the relevant response appeared filled in black, and the remaining three, in white (see ***Figure 1B***).

#### Procedure

The paradigm used in the fourth experiment is displayed in ***Figure 1B***. Trials started with the finger-tapping task, following the same timing parameters (tapping frequency and number of taps) as in Experiments 1–3, but without presenting any S-R mappings. After the three reset signal taps, participants saw a response cue indicating the required key press. The cue remained on the screen until the participants’ response or up to a maximum of 3000 ms.

Participants completed four 24-trial blocks, two using the index fingers for the finger-tapping task, and two using the middle fingers. The relevant finger-tapping modality was indicated at the beginning and in the middle of each block. Within blocks, we randomized the response required by the cues (left index, right index, left middle, right middle finger), and in consequence, the response set overlap condition (overlapping, non-overlapping response sets). Block order was pseudorandomized. Overall, 48 trials were collected per experimental condition.

Participants completed three practice sessions: one with the response cues alone, another with the finger-tapping task, and a final session with the combined dual task.

#### Data analysis

We followed the same criteria as in Experiment 1 to exclude participants and trials. Data from ten participants were discarded. Within participants, an average of 6% of trials was excluded. The percentage of excluded trials was similar in the non-overlapping (*M* = 6%, *SD* = 5%) and overlapping conditions (*M* = 5%, *SD* = 5%), *t*(81) = 0.98, *p* = 0.329, *Cohen’s d* = 0.11s.

We ran paired-sample *t*-tests contrasting error rates and RTs between non-overlapping and overlapping trials. Since one of our hypotheses predicted equivalent means between response overlap conditions, we also conducted two Bayesian paired-sample *t*-tests (Dienes, 2014). We interpreted *BF*_01_ above three as moderate evidence for the null hypothesis (Jeffreys, 1939).

### Results

**Figure 6:**
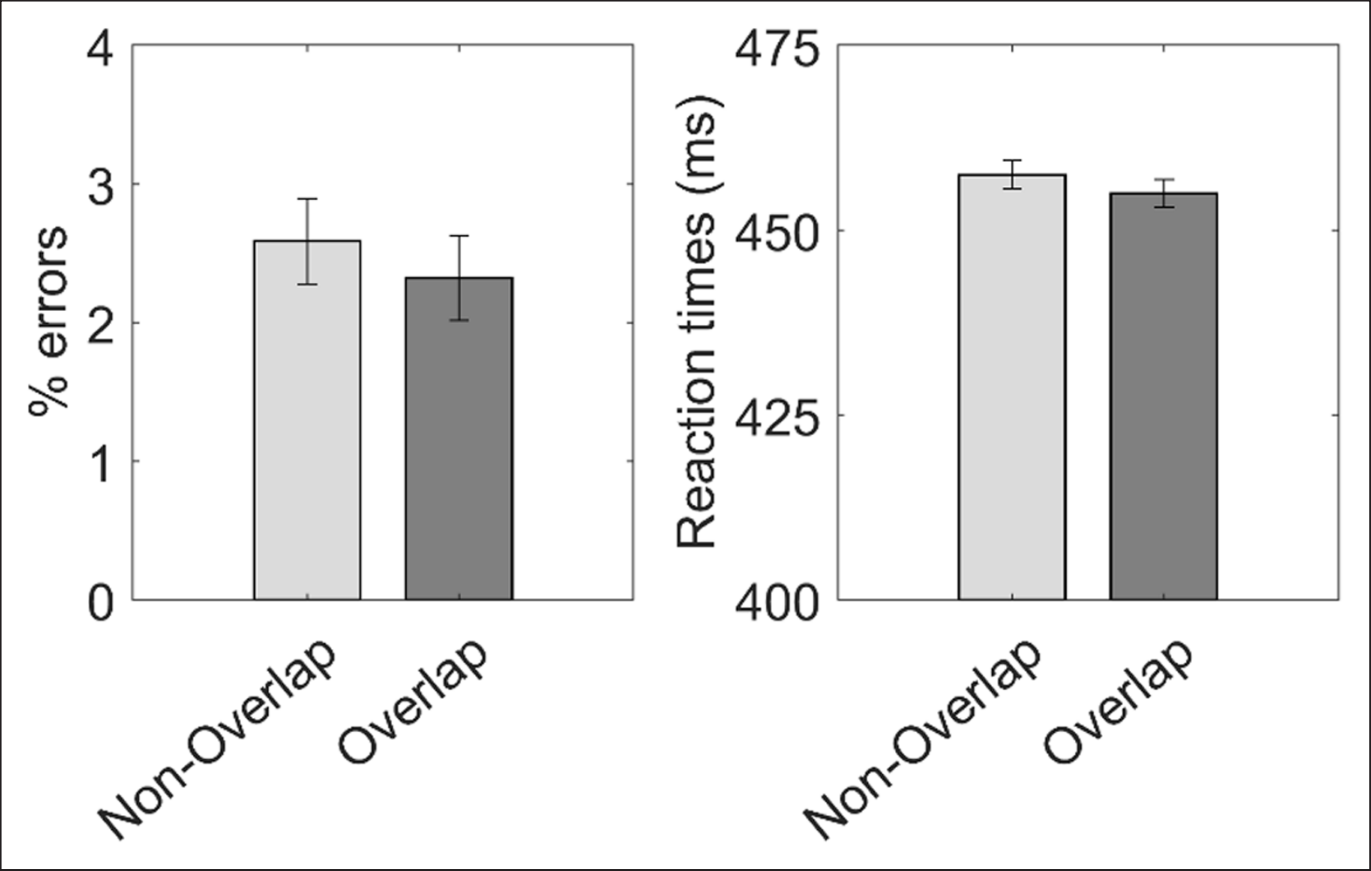
*Results from Experiment 4*: Mean error rate (left panel) and RT (right panel) in the two response overlap conditions. Error bars display 95% confidence intervals.

Mean error rates and RTs in the two response overlap conditions are displayed in ***Figure 6*** and Supplementary Table 4. Paired sample *t-*test showed no significant differences between response overlap conditions in either error rate, *t*(81) = 0.86, *p* = 0.393, *Cohen’s d* = 0.10, nor in RT, *t*(81) = 1.35, *p* = 0.181, *Cohen’s d* = 0.15. Bayesian *t-*test, conducted to confirm these null results, provided moderate evidence supporting equivalent means between conditions (error rate: *BF_01_* = 5.76; RT: *BF_01_* = 3.43).

We further analyzed tapping accuracy, delay, and variability (mean and standard deviation across conditions are shown in Supplementary Table 4). We did not find differences between conditions regarding tapping accuracy, *t*(81) = 0.82, *p* = 0.417, *Cohen’s d* = 0.09, and delay, *t*(81) = –0.82, *p* = 0.410, *Cohen’s d* = –0.09. Participants tapped significantly less variable in the overlapping condition, *t*(81) = –2.20, *p* = 0.030, *Cohen’s d* = –0.24.

### Discussion

This fourth, control experiment aimed to confirm or discard alternative accounts of the findings obtained in Experiment 1–3, like fatigue or negative priming. Frequentists and Bayesian evidence supported the absence of a response overlap effect in the absence of task preparation. Consequently, these results support that the effect found in the previous experiments was mediated by preparatory mechanisms – and not task-unspecific motor processing.

In this dataset, we found more rhythmical tapping in the overlapping condition. Since the participants were unaware of each trial’s overlapping condition (i.e., the cue was unpredictable), the interpretability of this result is uncertain. Nonetheless, a better, more rhythmical finger-tapping in overlapping trials should have boosted the impact of this condition. Consequently, it is unlikely that the reported null results were associated with a disengagement from the motor task in overlapping trials.

## General discussion

A substantial body of evidence stresses the quick transformation of novel task representations from a declarative into a procedural format (Demanet et al., 2016; González-García et al., 2020, 2021; Liefooghe et al., 2012, 2013; Liefooghe & De Houwer, 2018; Meiran et al., 2015a, 2015b; Meiran & Cohen-Kdoshay, 2012). Nonetheless, the cognitive processes mediating this transformation are uncertain to date (Formica et al., 2020, 2021; González-García et al., 2020, 2021). In this work, we interrogated a candidate mechanism: motor simulation (Grush, 2004; Jeannerod, 2004, 2001). Covertly activating the newly instructed responses could lead to the emergence of an action-based task code in the absence of previous overt performance, a rational motivated by both theoretical (Brass et al., 2017; Moran & O’Shea, 2020; Ruge & Wolfensteller, 2010) and empirical reasons (Theeuwes et al., 2018).

In a series of online experiments, we manipulated the availability of motor representations during novel mapping preparation. We hypothesized an impairment on performance when the mappings’ relevant response sets were already engaged by a dual finger-tapping task (Stevens, 2005). This prediction was robustly replicated across three datasets. Despite its ubiquity, we disentangled the response set overlap effect from general dual-task costs and purely motoric accounts. Critically, we additionally manipulated two proceduralization-related variables, task preparation (Liefooghe et al., 2013) and mapping novelty (Cole et al., 2018), to clarify whether we were tapping into this stage of novel instruction processing. Nonetheless, the overlap effect did not interact with these variables. Our task preparation manipulation led to an ambiguous trade-off effect on performance, questioning its validity. Nonetheless, task novelty successfully modulated behavior, with practice improving mapping implementation. The response set overlap effect was, however, insensitive to this variable. This null result suggests that while motor simulation may indeed be used during general task setting, its involvement is not specifically linked to novel task proceduralization, as we hypothesized.

Our null findings may relate to the abstract nature of novel tasks’ procedural representations (Cole et al., 2013; Meiran et al., 2017). It has been recently shown that novel mapping implementation is disrupted by concurrent verbal demands (van’t Wout et al., 2013; van’t Wout & Jarrold, 2020) or increasing the declarative working memory load (Formica et al., 2020). These results stress the role of a verbal component during instructed performance, in line with the idea that more abstract new task sets could require from verbal rehearsal to proper maintenance (Cragg & Nation, 2010; Kompa & Mueller, 2020). More directly related, it has been shown that the instructions’ procedural representations generalize across response modalities that are conceptually overlapping (Liefooghe et al., 2012). Taking all together, it may be the case that novel task proceduralization entails more high-level, abstract response representations than those engaged by our dual motor task. That would explain why the response set overlap effect was not specific for novelty.

Despite our results substantially deviated from our predictions, we still found robust evidence supporting that the covert activation of the relevant action representations is engaged by a preparatory mechanism, which generalizes across different cognitive contexts. Task preparation is a multidimensional process, acting at several hierarchical levels (De Baene & Brass, 2014). First, the procedural task representation must be activated. Nonetheless, this process differs depending on mapping novelty: new mappings require its assembly (via proceduralization; Brass et al., 2017) while for practiced ones, these representations can be retrieved from long-term memory (Mayr & Kliegl, 2000). Once the procedural representations are instantiated, they trigger a series of preparatory adjustments, biasing the processing across several downstream systems (Miller & Cohen, 2001; Sakai, 2008). Taking into account the nature of our manipulation, the preparation of the task’s motor component is the most likely stage affected by the overlap manipulation. Previous literature stresses that skilled performance is associated with a readiness state across the motor systems, allowing automatic response activation upon targets (Hommel, 2000). Importantly, recent evidence with electroencephalography recordings supports a similar mechanism for novel tasks, showing that novel instructions also lead to automatic response activation during preparation (Everaert et al., 2014; Meiran et al., 2015b). Taking into account that the advanced response reconfiguration seems to be common to both practiced and novel tasks, and the more general role of motor simulation in motor planning (Grush, 2004), blocking simulation during mapping encoding may have hindered this mechanism. In this regard, multiple components of the mappings’ actions were activated by our dual task: the effectors, the movement itself, and the movement’s kinesthetic contingencies. Several proposals emphasize the role of the latter – also known as action effects – for the goal-oriented control of behavior (Grush, 2004; Hommel et al., 2001). Future research disentangling the contribution of the individual action’s components would be of high relevance for current debates in the field.

Finally, our findings could also be relevant for theories of proactive cognitive control, a broader construct conveying task preparatory processes (Braver, 2012). Traditionally, proactive control is conceived as a domain-general function, exerting top-down influences in motor and perceptual interface systems (Miller & Cohen, 2001), implicitly assuming a serial, unidirectional processing chain (Norman & Shallice, 1986). While this theoretical view is parsimonious and straightforward, it does not incorporate the bidirectional and recursive influences between higher-level control systems and lower-level sensorimotor ones (Kilner et al., 2007; Summerfield & De Lange, 2014). In line with this perspective, we showed that the availability of motor representations may be necessary for task preparation – suggesting a role for the motor system in proactive control. Hence, our results advocate for a more embodied, action-oriented perspective on control processes. This partially overlaps with previous literature in other high-level cognitive domains, like semantics and language, which has evidenced that the involvement of sensorimotor representations is critical during information processing (Barsalou, 2008; Meteyard et al., 2012). Our data leaves open whether that view could also be extended to cognitive control processes. In this regard, we consider that more abstract task or goal representations are required to flexibly orchestrate behavior. However, these task sets may be built upon or enriched with action-based representation originated in the sensorimotor interface systems. Further behavioral and neuroimaging research would be key to shed some light upon this issue.

## Conclusion

In the present work, we addressed whether novel instruction proceduralization was based on the motor simulation of the upcoming task. Four experiments suggest that optimal task preparation may rely on motor simulation, but in a more general fashion – and not strictly in novel scenarios. We propose that the advanced reconfiguration of the task motor component is the candidate preparatory mechanism which may require from simulation. While we could not extract further insights on the proceduralization process, we suggest that the abstraction level of novel task sets should be taken into account in future research. Finally, we integrate our findings within broader theoretical accounts, emphasizing the role of the motor system in proactive cognitive control.

## Data Accessibility Statement

Each experiment preregistration, experimental stimuli, data, and analysis code are available on the Open Science Framework (*https://osf.io/qfshr/*).

## Additional File

The additional file for this article can be found as follows:

10.5334/joc.190.s1 Supplementary material. Supplementary Tables 1 to 4.
