# Supplementary material for: Exploring the Link between Novel Task Proceduralization and Motor Simulation
Source: J Cogn. 2021 Sep 27;4(1):57. doi: 10.5334/joc.190 (PMC8485871; doi:10.5334/joc.190)
Supplement: Supplementary material. — Supplementary Tables 1 to 4. [file joc-4-1-190-s1.pdf]

Supplementary Table 1

Mean and standard deviation from the S-R mapping and the finger-tapping tasks' dependent variables across conditions in Experiment 1.

	Control		Non-overlapping		Overlapping	
	M	*SD*	*M*	*SD*	*M*	*SD*
S-R mapping task						
Error rates	0.09	0.10	0.11	0.11	0.15	0.12
RT	749	275	765	274	795	292
Finger tapping task						
Motor task accuracy	-	-	89.9	10	90.3	9.6
Motor task delay	-	-	305	73	302	72
Motor task variability	-	-	156	36	157	37

Supplementary Table 2

Mean and standard deviation from the S-R mapping and the finger-tapping tasks' dependent variables across conditions in Experiment 2.

	Non-overlapping				Overlapping			
	Early deadline		Late deadline		Early deadline		Late deadline	
	M	*SD*	*M*	*SD*	*M*	*SD*	*M*	*SD*
S-R mapping task								
Error rates	0.12	0.09	0.08	0.05	0.15	0.09	0.11	0.08
RT	484	106	587	160	485	109	596	180
Finger-tapping task								
Motor task accuracy	92	7.3	91.7	8.2	92.2	7.7	91.7	8.3
Motor task delay	314	92	314	87	314	92	316	90
Motor task variability	154	38	154	35	153	38	155	36

Supplementary Table 3

Mean and standard deviation from the S-R mapping and the finger-tapping tasks' dependent variables across conditions in Experiment 3.

	Non-overlapping				Overlapping			
	Novel		Practiced		Novel		Practiced	
	M	*SD*	*M*	*SD*	*M*	*SD*	*M*	*SD*
S-R mapping task								
Error rates	0.07	0.06	0.06	0.05	0.09	0.07	0.08	0.07
RT	708	250	662	231	722	260	688	245
Finger-tapping task								
Motor task accuracy	93	5	92	6	93	5	92	6
Motor task delay	310	83	316	83	307	83	316	81
Motor task variability	154	36	155	35	151	67	156	34

Supplementary Table 4

Mean and standard deviation from the response cue and the finger-tapping tasks' dependent variables across conditions in Experiment 4.

	Non-overlapping		Overlapping	
	M	*SD*	*M*	*SD*
Response cue task				
Error rates	0.03	0.03	0.02	0.03
RT	457	70	455	70
Finger tapping task				
Motor task accuracy	91	5	92	6
Motor task delay	287	69	285	73
Motor task variability	153	40	156	40
